# From living metaphors in impactful dreams to sublime disquietude and sublime enthrallment

**DOI:** 10.3389/fpsyg.2026.1818552

**Published:** 2026-08-11

**Authors:** Don Kuiken, Ming-Ni Lee

**Affiliations:** 1 Department of Psychology, University of Alberta, Edmonton, AB, Canada; 2 Department of Counseling and Clinical Psychology, National Dong Hwa University, Hualien, Taiwan

**Keywords:** emergent meaning, impactful dreams, metacognition, poetic metaphor, sublime feeling

## Abstract

**Introduction:**

The continuing impact of spontaneously recalled dreams sometimes depends upon the same kind of unfolding semantic complexity that literary scholars attribute to poetic literary metaphors.

**Methods:**

We present the results of two online studies of impactful dreams (i.e., nightmares, existential dreams, and transcendent dreams) that reinforce this account.

**Results:**

In both Study 1 (*N* = 147) and Study 2 (*N* = 157), assessment of dream experience indicated that (a) existential dreams transition into sublime disquietude (inexpressible realizations, self-perceptual depth, disquietude) and (b) transcendent dreams transition into sublime enthrallment (inexpressible realizations; self-perceptual depth, reverence), while (c) nightmares, in contrast, transition into inflexible vigilance.

**Discussion:**

Because existential dreams and transcendent dreams support a type of metacognition that enables continued attunement to tension between the metaphoric (“is”) and literal (“is not”) truth-value of metaphoric dream images, these two types of impactful dreams may precipitate the epistemic—and aesthetic—significance of unfolding semantic resonance within the dream.

## 1 Introduction

Recent studies of dreams and literary reading indicate that an expressive-epistemic form of aesthetic response (called sublime feeling) emerges during impactful dreams (Kuiken, 2015, 2024) and during deeply engaged literary reading (Kuiken and Douglas, 2018; Kuiken and Sopčák, 2021). The terms chosen for sublime feeling in these studies (sublime disquietude, sublime enthrallment) reflect a basically Kantian conception, although subsequent scholarship (cf. Cazeaux, 2007) enables articulation of a conception of sublime feeling (a) that respects Kant’s primary objectives in his *Critique of the Power of Judgment*; Kant, 2009; hereafter *CPJ*; (b) that is based on a phenomenological conception of how aesthetic ideas facilitate the epistemic import of “living metaphor” (Ricoeur, 2004); and (c) that suggests empirical procedures for assessing the emergence of sublime feeling during impactful dreams and literary reading.

Within that theoretical framework, sublime feeling (a) is not only emotionally “moving” (Menninghaus et al., 2019) but also epistemically transformative (Kuiken, 2024) and (b) is not only a source of personal post-dream “insight” (Blagrove et al., 2019) but also of intra-dream movement toward an abstract-ontological grasp of the life-world (Tinguely, 2018). Also, relying on an established empirical typology (cf. Busink and Kuiken, 1996; Kuiken, 2015; Kuiken and Sikora, 1993), recent studies suggest that sublime disquietude and sublime enthrallment emerge during two types of impactful dreams (existential dreams and transcendent dreams, respectively). We propose that expressive imagery during existential and transcendent dreams is experienced neither as unidirectional metaphoric representation of post-dream waking concerns (Blagrove et al., 2019; Malinowski and Horton, 2015) nor as simulation of a seemingly “real” world (Nielsen, 1991; Solomonova et al., 2010). Instead, existential dreams and transcendent dreams involve attunement to the metaphorical “is” and literal “is not” that unfolds as iterative, self-relevant, and existential “living metaphor” (Ricoeur, 2004).

*Impactful dream types*. In earlier attempts to examine the significance of impactful dreams, Jung (1966) referred to “big” dreams (p. 117); States (1997) described dreams that have “magnitude” (p. 238); and others have isolated dreams that are exceptionally “intense” [e.g., Hartmann, 2008, p. 54]. It is difficult to determine whether impactful dreams are comprised of different types (“species”; cf. Hunt, 1989) or whether they occupy extreme points on a selected continuum (e.g., “dream-like fantasy,” Foulkes et al., 1966; “immersion,” Windt, 2021). Although there are taxometric procedures for systematically differentiating continuous from typological constructs (Borsboom et al., 2016), researchers studying impactful dreams have not availed themselves of that psychometric technology. In contrast, our rationale for adopting a typological approach takes account of evidence (Robert and Zadra, 2014; Schredl and Göritz, 2018) that linear definitions of hypothetical variables (e.g., the “intensity” of nightmares) (a) obscure incompatible forms of distress (e.g., sadness, fear); (b) reflect investigator presuppositions, rather than empirical evidence (e.g., the “absence” of sadness in nightmares); and (c) include attributes that extend beyond the manifest dream [e.g., disturbing dreams that awaken the dreamer [nightmares] and disturbing dreams [bad dreams] that do not (Zadra et al., 2006)].

Our procedures rely upon an established typological approach to categorial clarity (Beckner, 1959). In this approach, systematic comparison of dreams across an array of features provides the empirical basis for category articulation. A series of such taxonomic studies (Busink and Kuiken, 1996; Kuiken et al., 2006; Kuiken and Sikora, 1993; Lee and Kuiken, 2015) has revealed three types of impactful dreams. Nightmares involve fear/anxiety, harm avoidance, auditory anomalies, physical metamorphoses, and intense dream endings; existential dreams involve sadness/despair, separation and loss, movement inhibition (fatigue), unusual light/dark contrasts, spontaneous feeling shifts, and intense dream endings; and transcendent dreams involve ecstasy/awe, magical abilities, graceful movement, extraordinary sources of light, spontaneous shifts in perspective, and exceptionally vital dream endings. Mundane dreams, in contrast, generally lack these features.

*Lived experience during impactful dreams: preliminary studies*. A related series of studies explored whether the preceding impactful dream types disclose distinctive modes of lived experience during the dream. This possibility can be differentiated from the the notion that these dreams differentially alter post-dream experience (aftereffects). Evidence to date indicates that (a) the unfolding experience of nightmares involves lingering vigilance and distress (e.g., dream-induced anxiety, reluctance to resume sleep; Belicki, 1992a, 1992b; Blagrove et al., 2004; Köthe and Pietrowsky, 2001; Miró and Martínez, 2005; Nielsen and Levin, 2007); (b) the the unfolding experience of existential dreams involves self-perceptual depth [e.g., reaffirmation of existential convictions, sensitivity to aspects of life that are usually ignored; Busink and Kuiken, 1996; Kuiken et al., 2006; Kuiken and Nielsen, 1996; Kuiken and Sikora, 1993; Lee and Kuiken, 2015]; and (c) the unfolding experience of transcendent dreams involves spiritual revitalization [e.g., awakened spiritual potential, recovering the feeling of life; Kuiken et al., 2006; Lee and Kuiken, 2015]. These experiential changes may differ when nightmares occur in the context of other dream types [e.g., bad dreams; Tousignant et al., 2022], when there is extended opportunity for post-dream reflection on impactful dream experiences (Knudson and Minier, 1999), and so on. However, the present research addresses immediately lived experience during impactful dreams and, in particular, the immediate aesthetic-epistemic character of these dream types.

Pivotal in the present research is evidence that existential dreams and transcendent dreams (but neither nightmares nor mundane dreams) involve a distinctive form of metacognition (Busink and Kuiken, 1996; Kuiken et al., 2023; Kuiken and Sikora, 1993; Lee and Kuiken, 2015). Specifically, dreamers sense themselves from two points of view (e.g., “I became split into two parts; I was able to experience the dream world from either perspective”). This kind of duality not only departs from the single-mindedness of typical dreams (Rechtschaffen, 1978) but also differs from the concurrent awareness of—and perhaps control of—the dream scenario that are the allure of lucid dreaming (Windt and Voss, 2018). Nonetheless, this particular kind of metacognition is among several that have epistemic import (Dokic, 2012), including feelings of knowing (Goupil and Proust, 2023); tip-of-the-tongue experiences (Huebert et al., 2023); and moments of déjà vu (Cleary and Brown, 2021).

*The lived experience of metaphoricity during dreaming*. It is well established that, despite activation of the default mode cortical network during typical dreaming (Domhoff, 2018) and despite the increased accessibility of (a) associatively remote lexical items after REM sleep [cf. Stickgold et al., 1999] and (b) temporally remote memories after late night sleep (Picard-Deland et al., 2023), there remains some kind of continuity between the concepts and concerns represented in a person’s dreams and those independently represented during subsequent waking cognition (Domhoff, 2022). Although such continuity resists objective assessment (Malinowski, 2016, p. 120), it is evident even when assessment of these concepts and concerns is quite rigorous [e.g., Davidson and Lynch, 2012; Hoelscher et al., 1981]. Nonetheless, the semantic structure of relations between dreaming and post-dream cognition is an issue that remains unsettled. Available research indicates that (a) normatively weak lexical associations, ranging from hierarchical semantic relations to mere contiguity, are strengthened following REM sleep (Stickgold et al., 1999); (b) normatively weak associations to emotion words are strengthened when they are primed by presentation of those emotion words prior to REM sleep (Carr and Nielsen, 2015); (c) an “association” that captures the (schematic) gist of an array of semantically related words, ranging from part-whole to contextual relations, is “remembered” following REM sleep (Lewis and Durrant, 2011; Payne et al., 2009); and (d) there is greater post-REM readiness to identify modifier-modified (word-word) phrases that have the same root term [e.g., cottage cheese, cheesecake, Swiss cheese; Cai et al., 2009]. These results have been presented as evidence that memory traces activate relatively distant, uncommon, and novel semantic associations during—and immediately after—REM sleep.

It has been tempting (cf. Zadra and Stickgold, 2022) to generalize this spreading activation model to explain higher order (“creative”) cognitive phenomena. However, such generalization is problematic partly because, in the studies just mentioned, the weak associations strengthened during REM sleep range from those based on mere contiguity (word co-occurrence) to those predicated on basic semantic relations (i.e., nouns, verbs, adverbs, etc.). Generalization of the spreading activation model would be more compelling (and less obviously reductionist) if the weak associations under investigation differentially examined (a) categorial taxonomic relations (e.g., hypernyms, hyponyms, class co-membership); (b) part-whole relations (e.g., characteristic features, traits); (c) thematic relations (e.g., characteristic possessions, characteristic locations); and (d) figurative modifier-modified relations (Fogel et al., 2018).

Generalization of the spreading activation model would be even more compelling if the weak associations under investigation went beyond lexical-semantic relations to include higher level narrative and figurative semantic relations. Beyond evaluation of when dream cognition is “story-like” (Cipolli et al., 2020; Nielsen et al., 2001), effective generalization of the spreading activation model might well include examination of (a) narrative twists (e.g., an irregular episode within a thematically coherent episodic sequence) and (b) anomalous narrative outcomes (e.g., an unanticipated conclusion within a thematically coherent episodic sequence). Also, beyond evaluation of when lexical level semantic resonance occurs in a dream [e.g., a car and a bike as means of transportation; Rittenhouse et al., 1994], generalization of the spreading activation model might well include examination of the figurative forms, perhaps especially the metaphoric forms that, as hypothesized here, (a) are extended and elaborated during impactful dreams and (b) culminate in radical limit experiences involving sublime feeling (Arcangeli and Dokic, 2021).

*Metaphoric structures within impactful dreams*. Articulation of such narrative and figurative forms—and their aesthetic and epistemic import—motivates comparison of impactful dreams with pictorial (visual) and linguistic (literary) aesthetic objects. Basic theoretical background includes (a) the contrast between a “recognizable” pictorial object and its configurational “inflections” [“twofoldness”; Alloa, 2021; Wollheim, 1980, 1998] and (b) the contrast between the syntax and semantics of conventionally presented linguistic content and the linguistic “deviations” that constitute its “style” [Shklovsky; Berlina, 2017; Van Peer et al., 2021]. Accounts of pictorial inflections and linguistic style allow that, in some contexts (e.g., cinematic directorship, literary authorship), these stylistic inflections reflect the deliberate (agentic) intentions of a director or author (Steen, 2017; Wollheim, 1998). However, we will emphasize a form of reflexive intentionality that differs from these agentic forms. Specifically, we will emphasize attunement to (and a metacognitive sense of) the metaphoric “is” and literal “is not” within spontaneously unfolding dream imagery. This kind of metacognition departs from the “single-mindedness” of dreams, as well as from ordinary intra-dream self-reflection (Kahan and Sullivan, 2012; Lee and Kuiken, 2015). Instead, the distinctive form of metacognition found in existential and transcendent dreams (Busink and Kuiken, 1996; Kuiken et al., 2023; Kuiken and Sikora, 1993; Lee and Kuiken, 2015) is comparable to metacognition during the unfolding course of improvisatory performance (Colombetti, 2014; Gallagher and Gallagher, 2020; Sheets-Johnstone, 2012)—and it is comparable to the semantic source of what Ricoeur (2004) calls “living metaphor.”

We will focus on how this metacognitive tension supports the unfolding integration of categorial objects and their stylistic inflections. While it is possible to analytically distinguish conventional categorial objects from their stylistic inflections, it is important to emphasize their simultaneity and integration. Rather than the superimposition of stylistic inflections onto categorial content, we emphasize the co-constitution of a categorial object within the interactive nexus of conventional categorial and stylistically inflected representations. Rather than the modifying force of pre-established moods, intentions, or dispositions, this approach addresses the temporally extended interplay between content and style. To clarify this objective, what follows is a linguistic and then an oneiric example of such temporally extended interplay.

*Borges on Time.* The loci of such interplay are usually identifiable, as in the following excerpt from Borges (2000) essay, “A New Refutation of Time.” (This excerpt is useful here because it is neither a literary text nor a dream report.) In these lines, an understanding of time is metaphorically (and progressively) represented during reflective consideration of its moving, devouring, and immolating insistence:

Time is a river that carries me along,

and I am the river;

It is a tiger that devours me,

and I am the tiger;

It is a fire that consumes me,

and I am the fire.

In this excerpt, several local figurative features stand out. First, each line begins with a simple nominal metaphor (e.g., “Time *is* a river”). Second, each nominal metaphor is followed by *elaborative* (i.e., explicative, perhaps even ampliative) modulation of an initial metaphoric vehicle (e.g., “…it [the river] carries me along”). Third, each nominal metaphor with its elaborative modulation is followed by *compound* metaphoric modulation involving a nominal first-person metaphor (e.g., “I am the river”) that has both the initial metaphoric vehicle (e.g., “a river”) and, indirectly, the initial metaphoric topic (e.g., “time”) as its metaphoric topics (e.g., “I am” river-like time).

And yet, beyond these individual figurative features, a higher order figurative structure is also evident. Although Borges originally wrote this passage as prose, its three-part “verse” structure (accentuated by the line breaks added here) supports (a) three separate first-person compound metaphoric modulations (“I am” [river-like] time; “I am” [tiger-like] time; “I am” [fire-like] time) and (b) an integrative first-person compound metaphoric modulation (“I am” [river-like, tiger-like, fire-like] time). Are the separate (iterative) and inclusive (integrative) effects of these metaphoric modulations independently evident in the response of actual readers? Some empirical evidence indicates that they are. In an empirical study of young adult readers, Kuiken and Douglas (2018; Study 1) found that the separate (iterative) first-person compound forms (“I am [river-like] time”; “I am [tiger-like] time”; and “I am [fire-like] time”) prompted reports of *inexpressible realizations* (e.g., “I sensed something that I could not find a way to express”). They also found that the inclusive (integrative) compound form (“I am” [river-like, tiger-like, fire-like] time) distinctively precipitated inexpressible realizations with a concomitant sense of release. The latter result is consistent with informal observations (Gendlin, 1997) that such an ineffable realization is a “felt shift” accompanied by a momentary “sense of release” as though there is a subtle “letting go,” a subdued and softened “Oh, that’s there.” This sense of release plausibly marks a moment of sublime feeling.

*The hotel dream*. Comparably compounded metaphoric modulations are characteristic of impactful dreams. In the following prototypic existential dream (taken from Kuiken, 1995, p. 136), a three-episode structure (analogous to the Borges three-verse structure) progressively—and metaphorically—discloses the dreamer’s felt sense of the coercive character of inauthentically intimate relationships.

[I was in] a hotel in southern Alberta or someplace. I was traveling by myself, I think, and I remember worrying about rapists in the hall and this sort of thing. I remember thinking, “Well, I’ll have to be brave because I’m by myself.” So, I took this room in a secluded area of the hotel … and anyway it seemed to work out. // And then this hotel seemed to be in France. My family was with me, and we were all in a room together. We were packing to leave. I was very organized; I had all of my stuff ready to go … My family was very disorganized and I was having to help them. I did not want to. I thought, “Well, they can do it themselves; I’m not responsible for their packing.” But it was almost impossible not to help them because I needed another bag or two and I had things stored in a particular drawer and they had dumped all their stuff in there, too. So, in order for me to get this packing done, I had to help them anyway // [Then I think I had gone off on my own for a while] and I came back [to the hotel again.] I got a phone call, an overseas phone call from my Dad … He had gotten a doctor’s report, and the doctor said that he [had an ailment that] would never heal. And I had plans about my whole family moving to France … but he just told me how sick he was and that he would never heal. And there was some stupid person on the phone … some practical sounding person, who was sticking her nose in there. I kept telling her to shut up … and I was really upset and crying very hard. My Dad said that he wanted to talk to my Mom. So my Mom came to the phone [and she thought that it wasn’t practical to live in France]. She seemed to think that it was better to stay in Canada. I was surprised by her ability to say what was best for me … and I remember trying to talk her into it. I was overwhelmed by the fact that my father would never heal. I could not be with him and also stay in France. I woke up crying. I was just really sad. I felt this sadness just coming out of the bottom of my soul, from way down deep some place.

In this dream, three successive episodes (separated here by //) present semantically resonant portrayals of concern about coercive intimacy (worry about rapists in an Alberta hotel hallway; unwelcome assistance to filial fellow travelers in a hotel in France; aversion to a series of familiar voices who seem to know what is “best” for the dreamer). Although less simply than in the Borges lines that present compound metaphoric representations of “time,” this three-episode structure presents compound metaphoric representations of something like “the coercive character of inauthentically intimate relationships”.

Given persistent self-representation in dream narratives, this dream presents a series of three separate first-person compound metaphoric modulations (“I am here” [metaphorically] living with the possibility of rapists in the hallway; “I am here” [metaphorically] living in opposition to de facto filial collaboration; “I am here” [metaphorically] living with dispersed (parental) presumptions about what is best for me). As in the Borges lines, the result is a repetitive structure (a theme, a *leitmotiv*) that involves three separate first-person compound metaphoric modulations. In addition, an integrative higher order first-person compound metaphoric modulation is established by the series of first-person metaphoric modulations moving from (a) “I am here” [metaphorically] living with the possibility of rapists in the hallway to (b) “I am here” [metaphorically] living in opposition to de facto filial collaboration and then to (c) “I am here” [metaphorically] living with dispersed (parental) presumptions about what is best for me. This sequence is more clearly ampliative than the sequence of Borges lines, moving the dreamer toward a concluding dramatic and mournful sense of release: She concludes, “I felt this sadness just coming out of the bottom of my soul, from way down deep some place.” This moment of deep disquietude is a form of sublime feeling.

More complete articulation of the unfolding metaphoric structure of this dream would require the scrutiny derived from additional (open-ended) questions about nuances in the dream narrative (Petitmengin et al., 2019). Perhaps, however, the preceding analysis is sufficient to clarify how the entirety of this dream is analogous to the entirety of the highly structured Borges lines in the way it moves toward first-person metaphoric articulation of a theme and toward a poignant moment of sublime feeling—all *within the dream and prior to waking reflection*. We propose that this kind of metaphoric succession—and the progressive integration of metaphors of personal identification—constitutes the structure of sublime feeling within impactful (existential and transcendent) dreams. This kind of metaphoric modulation may characterize existential and transcendent dreams because these dream types plausibly support (a) attunement to (and a metacognitive sense of) the metaphoric “is” and literal “is not” of unfolding dream imagery (Kuiken, 2024; Kuiken et al., 2023); (b) generation of a succession of separate (iterative, thematic) first-person metaphoric modulations; and (c) integration of these first-person metaphoric modulations within a poignantly sublime dream ending.

*Emotion-centered conceptions of oneiric aesthetics*. There are precedents for the present proposal that dream images—perhaps especially the “intense” images of impactful dreams—possess the same “powerful” metaphoric configuration as occurs in poetry. One overly simple proposal is that the “positive emotions” that color transcendent dreams (e.g., ecstasy/awe) contrast with the “negative emotions” that color existential dreams (e.g., sad/downhearted). A more nuanced proposal, derived from studies of literary reading (Menninghaus et al., 2019), is that a poignant conjunction of positive and negative emotions makes impactful dreams especially “moving.” However, neither of these proposals is consistent with the available oneiric evidence, e.g., the absence of a poignant blend of positive and negative emotions in existential dreams, the absence of such a poignant blend within the fear and vulnerability that dominates nightmares (Hartmann, 2011; Kuiken, 2015; Robert and Zadra, 2014).

A model involving more complex figurative modulation of dream content is required. For example, Hartmann’s account of intense dreams (Hartmann and Kunzendorf, 2013) addresses the figurative (especially metaphoric) composites that represent powerful emotions. In his portrayal of how “central images” represent emotions, Hartmann borrowed T. S. Eliot’s description of the “objective correlative” of poetic verse, i.e., “a set of objects, a situation, a chain of events which shall be the formula of [a] particular emotion” (Eliot, 1921, p. 92). On one hand, Hartmann’s (2008) phrase (“central image”) suggests a *single* “picture-metaphor” that is “especially powerful, vivid, bizarre, or detailed” (e.g., his well-known tidal wave). On the other hand, Barcaro (2022), following Eliot’s wording more closely, suggests that impactful dreams involve a “*succession* of images, each of which is visually impactful” (italics ours; p. 5). As implied by the unfolding series of images in the Borges lines and the unfolding series of episodes in the Hotel Dream, there may be something aesthetically and epistemically distinctive in Barcaro’s emphasis on an unfolding succession of images.

*Epistemic conceptions of oneiric metaphoricity*. The metaphoric structures that resonate successively within impactful dreams (e.g., the Hotel Dream) suggest that, rather than emotion, the experience of impactful dreams depends upon the dreamer’s precarious grasp of a cumulative aesthetic and epistemic tension, specifically, a tension between the metaphoric “is” and literal “is not” within a succession of images. Existential dreams may evoke this metacognitive tension in the context of perceived discord and felt ineffectuality, resulting in sublime disquietude; transcendent dreams may evoke this ineffable tension in the context of perceived elevation and felt vitality, resulting in sublime enthrallment. Comparing the outcomes of existential and transcendent dreams with the effects of engagement with literary (poetic) verse has some empirical justification: sublime disquietude (as assessed in the present research) occurs during, for example, close readings of Paul Celan’s “Death Fugue,” while sublime enthrallment is reported after, for example, close readings of Percy Shelley’s “Mont Blanc” (Kuiken et al., 2012).

The present account locates oneiric sublime feeling within a hierarchy of figurative relations: (a) detectible image resonance; (b) detectible unidirectional metaphoricity; (c) detectible bidirectional metaphoricity; and (d) detectible metaphors of personal identification. We propose that levels within this hierarchy contribute quite differently to dream impact.

*Level 1: detectible image resonance*. So-called “discontinuities” in dream reports (Revonsuo and Salmivalli, 1995; Revonsuo and Tarkko, 2002) often involve weakly associated instances of a single categorial concept (e.g., a car and a bike, both as means of transportation; Rittenhouse et al., 1994; a person and a statue, both as human forms; Schweickert and Xi, 2010). During impactful dreams, dreamers may “sense” semantic resonance between such categorial “neighbors”—even without the syntactic structure that might, in another context, link them as modifying-modifier compounds (e.g., “statue people”) or as the topic and vehicle of nominal metaphors (e.g., “these people are statues”). Individuals participating in dream research may sense this kind of semantic resonance as indication that their dream was “impactful,” i.e., that it continued to influence their diffusely categorial thinking even after awakening.

*Level 2: unidirectional metaphoricity*. In studies of dreaming, conceptual metaphor theory has often been used to explain how a metaphoric vehicle is unidirectionally “mapped” onto a metaphoric topic (Blagrove et al., 2019; Lakoff, 1993; Malinowski, 2016; Malinowski and Horton, 2015). While vehicle attributes are often transformed prior to unidirectional mapping onto the topic, there is no consensus about how such transformations are derived (e.g., polysemy, analogy; Vega-Moreno, 2004). Moreover, while such transformations of the vehicle increase the salience of selected features of the *topic* that were not previously salient (Ortony, 1979), unidirectional mapping discloses nothing about the *vehicle* that was not already salient.

The immediately sensed “realism” of oneiric images supports unidirectional mapping. Empirical studies affirm that the sensed “realism” of images *during the dream* is usually displaced *after the dream* by the impression that they are “unrealistic,” “anomalous,” or even “bizarre.” Retrospectively “unrealistic” dream images invite—and perhaps demand—unidirectional metaphoric “mapping” of those “anomalous” or “bizarre” images onto post-awakening objects, events, emotions, and concerns (Blagrove et al., 2019; Blagrove and Lockheart, 2023; Malinowski, 2016; Malinowski and Horton, 2015).

*Level 3: bidirectional metaphoricity*. A more complex form of metaphoricity is evident during impactful dreams, especially during existential and transcendent dreams. This additional complexity is triggered by a distinctive form of metacognition. Judges’ ratings indicate that, even though REM and N2 dreams involve metacognition less frequently than wakefulness (Perogamvros et al., 2017), metacognition is nonetheless evident. It is most clearly evident when dreamers become aware of dreaming while dreaming, although intra-dream self-reflection is also common (Kahan and Sullivan, 2012; Lee and Kuiken, 2015). However, the issue here is not whether any (or many) forms of metacognition are evident in dream experience. We are concerned specifically with the type of metacognition that supports bidirectional metaphoric mapping.

Our basic proposal is that the dreamer’s capacity to sense the world of the dream from two points of view (Busink and Kuiken, 1996; Kuiken et al., 2023; Kuiken and Sikora, 1993; Lee and Kuiken, 2015; Kuiken et al., 2023) enables access to an epistemic tension that Searle (1979) argued is a fundamental aspect of metaphor comprehension (see also Giora et al., 2017). He proposed that metaphor comprehension involves dual attunement to (a) a *metaphoric* (A “is” B) assertion and (b) a complementary *literal* (A “is not” B) assertion. For example, to say that “all prayer is grief flying” brings attention to the metaphoric truth value of a statement (e.g., prayer [metaphorically] “is” grief) and simultaneously to its literal falsehood (e.g., prayer [literally] “is not” grief). This mode of metacognition involves detection of both aspects of this epistemic tension.

Detection of this epistemic tension, in turn, allows focus on the disclosive potential of oneiric metaphoric structures. A primary source of this potential is the distinctive bidirectionality (A “is” B *and* B “is” A) of poetic metaphors. While Lakoff and Turner (1989) dismissed bidirectional mapping, a tradition extending from Richards (1936) and Black (1962) to Fauconnier and Turner (2003) and Goodblatt and Glicksohn (2022) provides articulation of the creative potential of bidirectional metaphor comprehension. In contrast to unidirectional mappings (A “is” B; “Death is a fat fly”), the bidirectional epistemic import of the vehicle and topic (A “is” B *and* B “is” A; “Death is a fat fly” *and* “A fat fly is death”) leads to disclosure of *emergent* meanings (Reid and Katz, 2022). Emergent meanings are attributes of the topic and vehicle that were not salient for *either* category considered separately but that become salient for *both* categories during metaphor comprehension (e.g., *apropos* death and fat flies, both are forms of “lurking contamination”). There is now fairly secure evidence that emergent meanings derive from poetic metaphors (Becker, 1997; Terai and Goldstone, 2012; Tourangeau and Rips, 1991; Utsumi, 2005), although it remains unclear how the integration of (A “is” B) *and* (B “is” A) generates these emergent meanings.

To clarify this process, it is useful to begin with the domain interaction model of metaphor comprehension (Katz and Al-Azary, 2017; Tourangeau and Sternberg, 1982). The domain interaction model describes juxtaposition of a topic and vehicle that are both “semantically dense” (concrete, embodied, self-relevant; Westbury and Wurm, 2022) but that, at an abstract (hypernemic, taxonomic) level, “distant” from each other. Using this model, it is possible to identify attributes of the metaphoric vehicle that are sufficiently abstract to also be attributes of the metaphoric topic and, in that way, effect bidirectional metaphor comprehension. This model of metaphor comprehension suggests that the inclusiveness of an *abstract* ontological category may subsume two or more semantically dense *regional* ontological categories. The result is an abstract-ontological concept that is “inclusive” although (*pace* Kant) not “totalizing.” Both existential and transcendent dreams plausibly support not only Searle’s distinctive metaphoric/literal tension but also the emergent outcome of such metaphoric/literal interplay.

*Level 4: metaphors of personal identification*. A fourth important figurative relation within the present hierarchy is the extended self-relevant metaphoricity that provides a context for inexpressible realizations with felt release. The iterative modulations that characterize the extended metaphors of existential and transcendent dreams (e.g., in the Hotel Dream) generate an unfolding sense of a meaning-finding self that Cohen (1999) calls metaphors of personal identification. Consistent with Cohen’s account, there is fledgling evidence that engagement with nominal metaphors facilitates recognition of subsequent personifying metaphors (Dorst, 2011) and that the cumulative effect of recurrent personifying metaphors is a sense of self as the author of unfolding metaphor extension and explication (Bruhn, 2011). As an overly simple example, in response to “All prayer is grief flying,” extended metaphoric modulation contributes to tacit endorsement of the following locutions: “I am [the ascent of] prayer”; “I am [the anguish of] grief.” The progressive integration of compound metaphors of personal identification—and their poignantly sublime conclusion during, for example, the Hotel Dream—contrasts with the unidirectional metaphoric representations that characterize retrospective attempts to identify waking concerns (Blagrove et al., 2019; Malinowski and Horton, 2015). The abstract ontological effects of progressive thematic integration during the dream differ from the individual personal insights derived from retrospective dream reflection (Blagrove and Lockheart, 2023).

### 1.1 The abstract ontological limits of sublime feeling

Articulating a theory of the oneiric movement toward metaphor bidirectionality and metaphors of personal identification motivates close comparison with Kant’s (2009) discussion of the radical limit experiences that constitute sublime feeling. He proposed that interplay between a succession of sensible “aesthetic ideas” and insensible “rational concepts” move toward “symbolic” (metaphoric) approximation of an abstract-ontological concept. Husserl (1970, 1973) similarly described how interplay between a succession of intuitable (sensible) past, present, and anticipated “moments” (adumbrations) move toward “as if” representation of an abstract-ontological type. Ricoeur (2004) elaborated the Kant/Husserl framework to describe how this progressive interplay contributes to “living” metaphoric regeneration of abstract-regional ontologies—without acquiescence to transcendental “totalities.”

*Symbolic hypotyposis*. In Kant’s account, open attention to a succession of “aesthetic ideas” initiates “spontaneous” *movement toward* “totalizing” concepts (Tinguely, 2018). Kant argues that it does so by initiating a form of metaphoricity through which aesthetic ideas “approximate” rational conceptions of the “world as a whole.” He argues that the movement (*Schwung*) of the productive imagination toward totalizing concepts entails “symbolic” presentations that are “in accordance with an analogy” (*CPJ* 5: 352). Such “symbolic hypotyposis,” he says, is pivotal when analogy offers the only way to exhibit (or present) rational concepts. Because Kant’s theory of symbolic hypotyposis resembles Aristotle’s theory of metaphor, it is possible to construe his account as a general theory of metaphor. However, Pillow (2001) more specifically proposed that Kant’s theory of symbols resembles the “interactionist” theories of poetic metaphor offered by Richards (1936), Black (1962), and others (Goodblatt and Glicksohn, 2022). Clarification of this theoretical possibility follows a path leading from Kant’s discussion of symbolic hypotyposis to Ricoeur’s discussion of “living metaphor” (Geniusas, 2024). Toward the end of this theoretical path, Ricoeur (2004) presented living metaphor as.

… a mode of discourse that functions at the intersection of two domains, metaphorical and speculative. It is a composite discourse, therefore, and as such cannot but feel the opposite pull of two rival demands. On one side, interpretation seeks the clarity of the [abstract ontological] concept; on the other, it hopes to preserve the dynamism of meaning that the [abstract ontological] concept holds and pins down. This is the situation Kant considers in the celebrated paragraph 49 of the *Critique of the Faculty of Judgment* … [The interplay] in which imagination and understanding engage assumes a task assigned by the Ideas of reason, to which no [abstract ontological] concept is equal. But where the understanding fails, imagination still has the power of ‘presenting’ the Idea (*Darstellung*). It is this ‘presentation’ of the Idea by the imagination that forces conceptual thought to *think more*. Creative imagination is nothing other than this demand put to [abstract ontological] conceptual thought. (p. 303).

Ricoeur (2004) goes on to describe (a) how comprehension of a poetic metaphor entails “discourse [that is] turned back upon itself and spurred on by [another] metaphorical utterance”; (b) how such discourse is iteratively extended (perhaps indefinitely) to constitute a poetic theme; and (c) how poetic thematization approximates (without attaining) abstract ontological determination. Such iteratively extended and unfolding thematization does not simply reduce poetic interpretation to the identification of abstract ontological concepts but, rather, moves toward further metaphoric modulation that persistently—but always inadequately—approximates abstract ontological concepts. Although it is difficult to investigate the chained images that give epistemic import to the themes emerging from extended metaphors, doing so is pivotal in studies of how we comprehend the unfolding metaphoricity of a literary text (Crowell, 2005; Natanson, 1998), the unfolding metaphoricity of the Borges lines, or the unfolding metaphoricity of the Hotel Dream.

### 1.2 Two forms of sublime feeling

In *CPJ*, Kant described symbolic representation of a radical metacognitive tension that emerges during the progressive interplay between (sensible) aesthetic ideas and (supersensible) rational concepts. His account of the metaphoricity of sublime feeling is mostly restricted to a family of feelings that suggest enthrallment: respect, reverence, astonishment, admiration (*CPJ* 5: 246). Missing is reference to a family of feelings that suggest disquietude: angst, finitude, dread, unease. However, consistent with post-Kantian poetry and scholarship, the challenge is to contrast these two forms of sublime feeling while remaining faithful to the extended, unfolding, and “living” metaphoricity within the epistemic interplay between aesthetic ideas and abstract ontological concepts.

*Sublime disquietude*. To characterize sublime disquietude, we follow Ratcliffe (2021); Ratcliffe et al. (2014) account of how abstract ontological “absence” hovers between (a) perceived exclusion from possibilities for living that are no longer “mine” but remain accessible to others (e.g., feeling safe, knowing affection) and (b) perceived exclusion from possibilities for living that are due to inherently human (“totalizing”) limitations (e.g., self-determination, meaningful commitments). Ontological absence is sensed as a “loss” of or “doubts” about possibilities for living that are presented by an abstract ontological conception of what is inherently human.

Ontological absence is suggested by studies (Kuiken et al., 2006; Lee and Kuiken, 2015) indicating that the post-awakening effects of existential dreams include existential doubt (Batson and Schoenrade, 1991), e.g., “…doubt became an important part of what it means to be honest about my existential concerns.” Also, the self-perceptual depth that consistently is reported following existential dreams (Busink and Kuiken, 1996; Kuiken et al., 2006; Kuiken and Nielsen, 1996; Kuiken and Sikora, 1993; Lee and Kuiken, 2015) includes items that are not only self-relevant (e.g., “… I became sensitive to aspects of my life that I usually ignore”) but also relevant for humans in general (e.g., “… my sense of life seemed less superficial”). Consequently, the index of sublime disquietude used in the present research incorporates the interactive combination of rated disquietude, rated self-perceptual depth, and rated inexpressible realizations.

*Sublime enthrallment*. In contrast, to characterize sublime enthrallment, it is useful to consider the intimations of abstract ontological intersubjectivity that are sensed during (a) non-utilitarian attunement to reciprocal intentionality within a dichotomous relationship (*Ich und Du*; Buber, 1971) and (b) non-utilitarian attunement to the “inner subjectivity” of beings in general (i.e., “totalizing” intersubjectivity). Evidence of abstract ontological intersubjectivity is also provided in a recent study of independent dream character and dreamer subjectivity during lucid dreaming (Zadra and Green, 2025) and in studies (Kuiken et al., 2006; Lee and Kuiken, 2015) indicating that the post-awakening effects of transcendent dreams include the apprehension of distributed liveliness, e.g., “… I had the sense that everything around me was somehow alive.” Also, the self-perceptual depth that is reported consistently after transcendent dreams (Kuiken et al., 2006; Lee and Kuiken, 2015) reflects not only poignant self-referential “depth” (e.g., “… I became sensitive to aspects of my life that I usually ignore”) but also the disclosure of “depth” in the experience of humans more generally (e.g., “… my sense of life seemed less superficial”). Consequently, the index of sublime enthrallment used in the present research incorporates the interactive combination of rated spiritual revitalization, rated self-perceptual depth, and rated inexpressible realizations.

### 1.3 The present empirical studies

In the two studies described below, we expected that, in contrast to nightmares and mundane dreams:

- H_1_: Existential dreams will transition into sublime disquietude, i.e., the interactive combination of inexpressible realizations, self-perceptual depth, and disquietude (e.g., feeling deeply ill-at-ease, being keenly disturbed).
- H_2_: Transcendent dreams will transition into sublime enthrallment, i.e., the interactive combination of inexpressible realizations, self-perceptual depth, and reverence (e.g., felt inner freedom, awakened spiritual potential).

Because nightmares lack the level of metacognition that is evident in existential and transcendent dreams, we also expected that, unlike existential and transcendent dreams:

- H_3_: Nightmares will transition into persistent pre- and post-awakening vigilance (e.g., dream-induced anxiety, reluctance to resume sleep).

## 2 Methods (study 1)

### 2.1 Participants

Participants were selected from students taking introductory psychology at the University of Alberta. Eligible participants were those who reported during mass testing that they frequently remembered dreams and had recently experienced dreams that affected their waking thoughts and feelings (at least once per month during the preceding 12 months). In addition, they had either (a) experienced significant loss or trauma (within the preceding 6 months, the preceding 6–24 months, or the preceding 3–7 years) or (b) had never experienced significant loss or trauma or had experienced only minor loss or trauma during the preceding 7 years. Results for the effects of loss and trauma, which will not be presented here, were reported in a separate publication (Kuiken et al., 2023).

One-hundred and seventy-eight students participated in this study for partial course credit. After excluding those who did not complete both research sessions or meet the inclusion criteria for loss and trauma, we analysed the data from 147 participants (61% female, 39% male; mean age = 19.8 years; ethnic background: 56% Euro-North American and European, 32% East and South Asian, 12% Other). Each of these 147 participants provided complete condition-specific loss and trauma information and sufficient information about their dreams to differentiate nightmares, existential dreams, transcendent dreams, and mundane dreams. (Ethics approval was provided by the University of Alberta Faculty of Arts, Science, and Law Research Ethics Board; Protocol ATEE 454.)

### 2.2 Procedures

At the beginning of an initial laboratory session, participants were informed of the nature of the study, assured of the anonymity and confidentiality of their participation, and asked for written and informed consent. Participants then completed the Loss/Trauma Questionnaire (LTQ), which enabled detailed determination of their loss and trauma histories, and a brief Sleep/Dreams Questionnaire (SDQ), which assessed the frequency of recent dream events (e.g., sleep paralysis, recurring dreams, dream recall). Then participants were told how to access the online research materials from their homes. Specifically, they were told to record, immediately after awakening, the first impactful dream that they experienced after the initial session (i.e., the first dream that seemed at least as impactful as their most impactful dream during the preceding 4 weeks). Participants were allowed up to 1 month to report an impactful dream.

During an online session immediately after awakening from an impactful dream, participants were asked to provide a narrative account of their impactful dream (Impactful Dreams Report; IDR), as well as to complete the related Impactful Dreams Questionnaire (IDQ) and Post Dream Questionnaire (PDQ). Later that day, they completed several other questionnaires; those findings were previously reported (Lee and Kuiken, 2015).

### 2.3 Materials

Impactful dream report (IDR). The IDR (Lee and Kuiken, 2015) was designed to obtain a narrative report of the participant’s dream experience. Participants were asked to describe in as much detail as possible: (a) all dream objects, places, characters, and events; (b) the entire sequence of actions and events; (c) their moment-to-moment thoughts and feelings; and (d) any unusual, incongruous, or implausible dream thoughts, feelings, objects, places, characters, or events.

Impactful dreams questionnaire (IDQ). A 26-item version of the IDQ, based upon previous studies of impactful dreams (Busink and Kuiken, 1996; Kuiken et al., 2006; Kuiken and Sikora, 1993), was used to differentiate three types of impactful dream types from mundane dreams. Ratings were provided using a scale that ranged from (0 = Not at all true [false]) to (4 = extremely true).

*Impactful dream types*. In a first step, dream types were identified based on assessment of their similarities to the rating profiles that distinguished nightmares, existential dreams, transcendent dreams, and mundane dreams in previous studies (Busink and Kuiken, 1996; Kuiken et al., 2006; Kuiken and Sikora, 1993). Specifically, squared Euclidean distances between the 26-item rating profile of each dream and the 26-item rating profiles of each dream type were determined, providing a basis for comparing the similarities between each reported dream and the overall rating profiles of each dream type. For instance, if the overall rating profile of a reported dream was most similar to the transcendent dream profile, this dream was classified as a transcendent dream. Among the 178 participants who provided complete loss, trauma, or traumatic loss information, 147 individuals also provided sufficient information to differentiate nightmares, existential dreams, transcendent dreams, and mundane dreams. Among the 147 participants in this final sample, there were 36 nightmares, 43 existential dreams, 33 transcendent dreams, and 35 mundane dreams.

*Impactful dream features*. An index of each impactful dream type was created from the average ratings on items used to assess the features of each dream type that have consistently been found type-specific across previous studies (Existential Dream Features [9 items; Cronbach’s *α* = 0.69]; Transcendent Dream Features [10 items; Cronbach’s *α* = 0.64]; Nightmare Features [8 items; Cronbach’s *α* = 0.71]). This strategy ensured that each dream type could be evaluated using an analogous array of features (feelings/emotions, narrative themes, movement characteristics, sensory anomalies, spontaneous transformations, effectuality/ineffectuality, and concluding affective tone).

*Existential dream features*: (1) “sad” and (2) “downhearted” at “the most intensely emotional moment of this dream”; (3) narrative theme, “My dream involved separation, rejection, or loss”; (4) movement characteristics, “In my dream I felt tired, weak, or unable to move”; (5) sensory anomalies, “My dream involved especially vivid contrasts between light and darkness”; (6) spontaneous transformations, “In my dream I experienced the spontaneous emergence of clear and distinct feelings”; (7) effectuality/ineffectuality, “In my dream I was successful in attaining my goals” (reverse scored); (8) concluding affective tone, “My feelings became especially intense just before I awakened”; (9) other, “In my dream I felt like crying—or I actually cried”.

*Transcendent dream features*: (1) “ecstatic” and (2) “in awe” at “the most intensely emotional moment of this dream”; (3) narrative theme, “My dream involved characters (including myself) with exceptional or even magical abilities (e.g., the ability to heal, to fly, to “know” others’ thoughts)”; (4) movement characteristics, “At times my movements in the dream were vigorous and energetic” and (5) “At times my movements in the dream were exceptionally well balanced and graceful (e.g., I was floating, gliding or flying)”; (6) sensory anomalies, “My dream involved unusual forms of or sources of light”; (7) spontaneous transformations, “In my dream I experienced a distinct shift in visual perspective (e.g., suddenly seeing things from above, suddenly seeing things as another character saw them)”; (8) effectuality/ineffectuality, “In my dream I was successful in attaining my goals”; (9) other, “My dream involved sensations of spreading warmth”; (10) other, “I felt exceptionally vital, energetic, and alive”.

*Nightmare features*: (1) “scared” (2) “terrified” and (3) “afraid” at “the most intensely emotional moment of this dream”; (4) narrative theme, “In my dream I repeatedly tried to avoid harm to myself or others”; (5) movement characteristics, “At times my movements in the dream were vigorous and energetic”; (6) sensory anomalies, “My dream involved unusual and disturbing sounds”; (7) spontaneous transformations, “In my dream there were sudden changes in the physical appearance of persons, places, or things (e.g., one person becoming another person, one place becoming another place)”; (8) concluding affective tone, “My feelings became especially intense just before I awakened.”

*Post-dream questionnaire (PDQ)*. The PDQ included several scales used to develop indices of the primary dependent variables: (a) Self-Perceptual Depth, a 6-item scale, e.g., “I became sensitive to aspects of my life that I usually ignore” (Kuiken et al., 2012; Cronbach’s *α* = 0.73); (b) Inexpressible Realizations, a 3-item scale, e.g., “I sensed something that I could not find a way to express” (Kuiken et al., 2012; Cronbach’s *α* = 0.86); (c) Disquietude, a 3-item scale, e.g., “I felt intensely disturbed” (Kuiken et al., 2012; Cronbach’s *α* = 0.80); (d) Spiritual Revitalization, a 3-item scale, e.g., “I felt a new sense of my spiritual potential” (adapted from Lee and Kuiken, 2015; Cronbach’s *α* = 0.69); and (e) Pre- and Post-Awakening Vigilance, an array of items previously reported by Belicki (1992a, 1992b), e.g., “I was afraid to fall asleep again” (Cronbach’s *α* = 0.69). Using the ratings on each of these scales, we created three indices of dream impact:

- *Sublime Disquietude* = SQRT(SQRT(SQRT((Inexpressible Realizations)*(Self-Perceptual Depth)*(Disquietude))))
- *Sublime Enthrallment* = SQRT(SQRT(SQRT((Inexpressible Realizations)*(Self-Perceptual Depth)*(Spiritual Revitalization))))
- *Pre- and Post-Awakening Vigilance* = MEAN(Pre- and Post-Awakening Vigilance)

### 2.4 Results (study 1)

#### 2.4.1 Aesthetically relevant features of impactful dreams

*Lived experience and impactful dreams*. We conducted three univariate analyses of variance with (a) Dream Type as the independent variable and (b) either Sublime Disquietude, Sublime Enthrallment, or Pre- and Post-Awakening Vigilance as the dependent variable. As expected (H_1_), for Sublime Disquietude, there was a significant Dream Type main effect, *F*(3,143) = 15.485, *p* < 0.001. Planned comparisons indicated that Existential Dreams were more likely to transition into Sublime Disquietude than were Mundane Dreams (*p* < 0.001) or Nightmares (*p* = 0.007). Also as expected (H_2_), for Sublime Enthrallment, there was a significant Dream Type main effect, *F*(3,143) = 17.038, *p* < 0.001. Planned comparisons indicated that Transcendent Dreams were more likely to transition into Sublime Enthrallment than were Mundane Dreams (*p* < 0.001) or Nightmares (*p* < 0.001).

Finally, as predicted (H_3_), for Pre- and Post-Awakening Vigilance, there was a significant Dream Type main effect, *F*(3,143) = 13.288, *p* < 0.001. Planned comparisons indicated that Nightmares were more likely to transition into Pre- and Post-Awakening Vigilance than were Mundane Dreams (*p* < 0.001) or Transcendent Dreams (*p* = 0.031). However, Existential Dreams were *also* more likely to transition into Pre- and Post-Awakening Vigilance than were Mundane Dreams (*p* < 0.001). Apparently, the persistant effects of Existential dreams include both Sublime Disquietude and Pre- and Post-Awakening Vigilance. This finding is compatible with the contrast between (a) absence-related depression (nightmares as involving unmitigated sympathetic ANS activation) and (b) absence-related melancholy (existential dreams as involving sympathetic ANS activation modulated by parasympathetic ANS activation) (Kuiken et al., 2023). Further conceptual and empirical examination of that contrast is crucial for the present project.

## 3 Methods (study 2)

### 3.1 Participants

Participants again were selected from students taking introductory psychology at the University of Alberta. Eligible participants were those who reported during mass testing that they frequently remembered dreams and had experienced dreams that affected their waking thoughts and feelings (at least once per month during the preceding 12 months). In addition, they had either (a) experienced significant loss, trauma, *or traumatic loss* (within the preceding 6 months, within the preceding 6–24 months, or within the preceding 3–7 years) or (b) had never experienced significant loss, trauma, or traumatic loss or had experienced only minor loss, trauma, or traumatic loss during the preceding 7 years. Results for the effects of loss, trauma, and traumatic loss were reported in a separate publication (Kuiken et al., 2023).

One-hundred and ninety-five students participated in this study for partial course credit. After excluding those who did not complete both research sessions or meet the inclusion criteria for loss, trauma, and traumatic loss, we analysed the data from 190 participants (72% female, 29% male; mean age = 20.6 years; ethnic background: 42% Euro-North American, 17% European, 26% East and South Asian, 15% Other). (Ethics approval was provided by the University of Alberta Faculty of Arts, Science, and Law Research Ethics Board; Protocol ATEE 454.)

### 3.2 Procedures

The procedures were very nearly the same as in Study 1, except for division of the loss group into two subgroups: (a) a non-traumatic loss group and (b) a traumatic loss group. In addition, the wording of some questions and rating scales was adjusted in an attempt to improve reliability. For the indices of each impactful dream type derived from the IDQ, the internal consistencies were modestly improved: Existential Dream Features [Cronbach’s *α* = 0.71]; Transcendent Dream Features [Cronbach’s *α* = 0.75]; and Nightmare Features [Cronbach’s *α* = 0.80]. Among the 190 participants who provided complete loss, trauma, or traumatic loss information, 157 individuals also provided sufficient information to differentiate nightmares, existential dreams, transcendent dreams, and mundane dreams. Among these 157 participants, there were 43 nightmares, 48 existential dreams, 22 transcendent dreams, and 44 mundane dreams.

### 3.3 Materials

*Central image intensity*. A judge trained in (the late) Ernest Hartmann’s dream research group (and blind to the dream typology presented here) scored this set of dreams on a 7-point scale for the “intensity” of each dream’s “central image” (Hartmann and Kunzendorf, 2013, p. 167–168), which he proposed is a marker of Eliot’s objective correlative. Ratings of the intensity of each dream’s central image was based on “how powerful, vivid, bizarre, and detailed the image seemed to be.”

### 3.4 Results (study 2)

*Lived experience and impactful dreams*. We conducted univariate analyses of variance with (a) Dream Type as the independent variable and (b) either Sublime Disquietude, Sublime Enthrallment, or Pre- and Post-Awakening Vigilance as a dependent variable. As in Study 1 (H_1_), for Sublime Disquietude, there was a significant Dream Type main effect, *F*(3,150) = 8.941, *p* < 0.001. Planned comparisons indicated that Existential Dreams were more likely to transition into Sublime Disquietude than were Mundane Dreams (*p* < 0.001) or Nightmares (*p* = 0.045). Also, as in Study 1 (H_2_), there was a significant Dream Type main effect, *F*(3,150) = 4.492, *p* = 0.005. Planned comparisons indicated that Transcendent Dreams were more likely to transition into Sublime Enthrallment than were Mundane Dreams (*p* = 0.017) or Nightmares (*p* < 0.001). The adjusted degrees of freedom in these analyses reflect deletion of a small group of participants who indicated careless responding on at least 2 of 3 instructed response items on the questionnaire used to assess loss, trauma, or traumatic loss (see Kuiken et al., 2023; see also Ward and Meade, 2023). Careless responding was not assessed on the IDQ.

Finally, as in Study 1 (H_3_), for Pre- and Post-Awakening Vigilance there was a significant Dream Type main effect, *F*(3,151) = 23.340, *p* < 0.001. Planned comparisons indicated that Nightmares were more likely to transition into Pre- and Post-Awakening Vigilance than were Mundane Dreams (*p* < 0.001) or Transcendent Dreams (*p* < 0.001). Also, Existential Dreams were more likely to transition into Pre- and Post-Awakening Vigilance than were Mundane Dreams (*p* < 0.001), reinforcing the possibility that the persistent effects of Existential Dreams include both Sublime Disquietude and Pre- and Post-awakening Vigilance. As indicated above, this (demonstrably replicable) phenomenon warrants further conceptual and empirical articulation (cf. Kuiken et al., 2023).

#### 3.4.1 Additional findings

*Central image intensity*. The preceding results reinforce the possibility that sublime feeling is an aesthetic outcome of Existential Dreams and Transcendent Dreams—but perhaps not Nightmares. This possibility is at odds with Hartmann’s (Hartmann and Kunzendorf, 2013) notion that the intensity of a central image in impactful dreams (including nightmares) represents the “objective correlative” of dream imagery and captures an image’s aesthetic potential. As expected, Central Image Intensity was positively correlated with Nightmare Characteristics, *r* = 0.42, *p* < 0.001, and with the interactive combination of Nightmare Characteristics and Pre- and Post-awakening Vigilance, *r* = 0.27, *p* < 0.001. However, Central Image Intensity was *not* significantly correlated with Existential Dream characteristics, Transcendent Dream characteristics, the interactive combination of Existential Dream characteristics and Sublime Disquietude, or the interactive combination of Transcendent Dream characteristics and Sublime Enthrallment. Hartmann’s single-image index of the objective correlative does not capture the progressive multi-image character of sublime feeling that occurs during Existential Dreams and Transcendent Dreams. Examination of this possibility may require nuanced description of individual dream narratives comparable to our portrayal of the Borges lines and the Hotel Dream. It may also require (open-ended) first-person exploration of the dream narrative using recently developed phenomenological methods (cf. Petitmengin et al., 2019).

## 4 General discussion

The results of the preceding studies confirm that existential dreams transition into sublime disquietude (H_1_); transcendent dreams transition into sublime enthrallment (H_2_); and nightmares transition into pre- and post-awakening vigilance (H_3_). This pattern (see especially Study 2) prompts reconsideration of when, if at all, nightmares generate poetic metaphors—and their aesthetic-epistemic effects (contra Barcaro, 2022; Hartmann and Kunzendorf, 2013). Instead, this pattern is consistent with our proposal that the transition into sublime feeling is precipitated by a distinctive form of metacognition specifically within existential and transcendent dreams (Kuiken, 2024; Kuiken et al., 2023). The level of metacognition within those two dream types may enable attunement to tension between the unfolding metaphoric and literal truth-value of resonant categorial dream objects. The felt sense of that tension in those two dream types may facilitate disclosure of the unfolding epistemic—and aesthetic—significance of successive bidirectional metaphoric images, culminating in the depth of sublime feeling (perhaps especially during the transition from sleep to wakefulness).

### 4.1 REM sleep neurophysiology

The present account identifies oneiric sublime feeling as a distinctive REM sleep carryover effect. Examining the neurophysiology of that effect is a challenge because the infrequent and aperiodic character of impactful dreams effectively precludes their observation within a sleep laboratory. A comparable methodological limitation prevails in studies of sleep paralysis, although a few laboratory case studies indicate that isolated sleep paralysis episodes occupy an intermediate position between the cortical and neuromuscular activation of wakefulness and the cortical activation and muscle atonia of REM sleep. The result is that the dreamer is “partially aware” of the sleep environment (Mainieri et al., 2021, p. 723). Similarly, we suggest, during existential and transcendent dreams, the dreamer is “partially aware” of dream images that seem metaphorically true and literally not true.

There is another reason to consider the comparison of impactful dreams and sleep paralysis episodes. Cheyne et al. (1999); (see also Cheyne and Girard, 2007) reported three types of sleep paralysis: (a) *intruder* episodes (multi-sensory hallucinations of a threatening figure); (b) *incubus* episodes (multi-sensory hallucinations of chest pressure, suffocation, and pain); and *vestibular-motor* episodes (illusory movement and out-of-body experiences). This tri-partite typology is analogous to the tri-partite typology of impactful dreams. Perhaps, then, nightmares, existential dreams, and transcendent dreams are muted or modified forms of the same tripartite psychobiological network that Cheyne et al. argue is the substrate of intruder, incubus, and vestibular–motor sleep paralysis episodes. If so, the REM sleep substrate of impactful dreams may require re-consideration. Rather than activation of the default mode cortical network that accompanies typical dreaming (Domhoff, 2018, 2022) and rather than the *alternating* activation of the default mode and executive control cortical networks that underlies sleep paralysis episodes, *coordinated* activation of the default mode *and* executive control cortical networks may constitute the substrate of existential dreams and transcendent dreams. Further conceptual articulation and empirical examination of the coordinated activation hypotheses is warranted (cf. Beaty et al., 2023).

Moreover, examination of the coordinated activation hypotheses may help to articulate the relations between (a) a phenomenological account of transitional moments of “sublime feeling” during existential and transcendent dreams and (b) a contrasting phenomenological account of transitional moments of “presence” during all three types of sleep paralysis (see Solomonova, 2018; Solomonova and Carr, 2022). Solomonova et al. emphasize an embodied and enactive cognitive science approach to cognition, depending especially on the late Husserlian framework provided by Varela et al. (1991), From that perspective, they emphasize the sensed “presence” of imaginal objects, places, and personae during sleep paralysis. Their emphasis on generic imagination contrasts with the focus here on productive imagination during the sublime feeling of existential and transcendent dreams (ala Ricoeur; cf. Geniusas, 2024).

#### 4.1.1 Specific implications

In general, the short scales used in the present research were minimally reliable, although that basic psychometric requirement should receive closer attention in revised scales for Inexpressible Realizations, Self-Perceptual Depth, Spiritual Revitalization, etc. On the other hand, while the formulas used to create indices of Sublime Disquietude and Sublime Enthrallment are appropriately non-linear and interactive, they also lead to nonlinearities that our square root corrections do not always address.

*Sublime disquietude and existentiality*. Studies of sublime feeling during existential dreams warrant comparison with empirical studies of cathartic response to tragic fiction (cf. Fuchs, 2018; Koopman, 2013; Kuiken and Sharma, 2013; Kuiken and Sopčák, 2021). In these efforts, it is important to step away from a purgative conception of catharsis in favor of something comparable to what Heidegger (1996) characterized as the “lived experience” of time: (1) the futural projection of an inevitable (but unpredictable) absence; (2) “prejudices” accumulated during a more nearly given than chosen past; and (3) the present sense of a “not yet” that is a person’s “ownmost” possibility. This discomfiting “not yet” can be experienced as an uncanny *Unheimlichkeit* in which the everyday living present becomes “close” but “foreign in its very proximity” (Blanchot, 1993, p. 45). Explication of this enigmatic closeness is consistent with some evidence from the present studies that links sublime disquietude with an empirical index of Existential Awareness (e.g., “I began to reconsider my existential convictions”). (These results are reported in the Supplemental materials.)

*Sublime enthrallment and spirituality*. Studies of sublime feeling during transcendent dreams warrant comparison with empirical studies of spiritual experience. Two recent projects used a dream-specific adaptation of the psychometrically well-crafted Mystical Experience Scale (MES; Hood and Francis, 2013). The Dreaming Mysticism Scale (DMS; Sears, 2015; Sears and Hood, 2016), like the MES, includes indices of extraverted and introverted mysticism that, not coincidentally, resemble the loss of self-boundaries subscale used in attempts to validate our index of sublime enthrallment. Explication of this enigmatic liveliness is consistent with some evidence from the present studies that links sublime enthrallment with an empirical index of Loss of Self-Boundaries (e.g., “My sense of self did not have a clear boundary”). (These preliminary results also are reported in the Supplemental materials).

#### 4.1.2 Implications for alternative procedures

*Various similarity indices*. The mode of cluster analysis on which the preceding impactful dream types are based groups (a) any two objects (reported dreams) that have a minimum distance value and (b) any two objects (across different dream clusters) that have a maximum distance value. Such distance-based clustering may derive from several distance estimates, including Pearson product–moment correlations, Euclidian distance coefficients, and cosine coefficients. The specific procedures used in the preceding studies invoked Euclidian distances squared to assess mutual similarities in their overall profiles of ratings (Bailey, 1994).

Ward’s method is hierarchical, i.e., it allows the identification of subgroups within a cluster, and the resulting clusters are mutually exclusive. The outcome of cross-cluster comparison, then, enabled identification of the analogous 8–10 specific attributes that most clearly differentiated dream types (across feelings/emotions, narrative themes, movement characteristics, sensory anomalies, spontaneous transformations, effectuality/ineffectuality, and concluding affective tone). That assessment of type centrality may, in turn, facilitate the next step in this series of studies: determination of network connections. Specifically, there are now bootstrap routines that can be used to identify network connections within a typology (Borsboom, 2022). These procedures may help to identify how (or whether) those analogous 8–10 specific dream attributes predict—and perhaps explain—cluster attributes that *depend upon* that subset of dream features.

*Various interaction models*. These analogous sets of dream attributes may also be used to explore the modes of dream engagement that distinctively predict the interactive combination of aesthetic-epistemic attributes that define sublime disquietude and sublime enthrallment. The formulae that define sublime disquietude and sublime enthrallment in the present studies (and in prior studies of literary reading; Kuiken et al., 2012) depend upon linear interaction regression models; they pointedly avoid simpler additive models in which the effect of one variable is independent of the value of another. We anticipated, for example, that the effects of inexpressible realizations on sublime disquietude would depend upon levels of disquietude.

This approach precludes the possibility that one component of the tripartite model (e.g., disquietude) would dominate the conception of sublime disquietude that was at stake. It should also be noted that linear predictive models—even with interaction terms—are especially apt in the examination of mediating variables. Such mediation, however, may support examination of the causal origins of dream outcomes (aftereffects) but it was important in the present project to retain the unfolding complexity of the ongoing impactful dream experience as exemplified by the Borges lines and by the Hotel Dream.

*Various indices of fusion*. The preceding interactive relations also facilitate examination of recent developments in the content analysis of impactful dreams. Judges in in a study by Kirberg and Windt (2026) rated transcribed dream reports and mind-wandering episodes using and *The Content Analysis of Bizarreness Scale* (Revonsuo and Salmivalli, 1995). The authors found that dream reports and mind-wandering episodes differed in ways that suggest use of these methods in future studies of impactful dreams. Specifically, they found that fused identity (e.g., a dream character resembling both a friend and a partner) was completely absent in mind-wandering reports—and unique to dream content. In contrast, abrupt discontinuity (abrupt topic changes or disappearances) was more prominent during mind-wandering. These findings reinforce our account of the first level of metaphor-like resonance, suggesting that recurrence of such resonance across a sequence of dream episodes may be the substrate of unfolding dream impact.

In this context, close consideration of nuanced dream anomalies invites (perhaps requires) a well-delineated list of types of “bizarreness,” the articulation of category assignment rules, and rules affecting ambiguous cases. As the Revonsuo and Salmivalli (1995) project demonstrates, this is not an inconsequential project—and the scoring details can be very demanding indeed.

#### 4.1.3 Some general methodological implications

By implication, it is important to press for articulation of the content categories that are grounded in “living” aesthetic/epistemic metaphors–and to identify moments of sublime feeling. This may not be the place to expect straightforward operational definitions of either typological or continuous constructs (Borsboom et al., 2016). Research areas pertaining to the aesthetic/epistemic effects of “living” oneiric or literary metaphoricity more likely begin with the kind of *connoisseurship* that provides *reliable* articulation (content validity) of the metaphoric structure of textual material comparable to that in (a) the Borges lines about time, (b) existential dreams (e.g., the Hotel Dream), (c) transcendent dreams (cf. the prototypic example reported in the Supplemental materials), and (d) creative lucid dreams (Štuikytė and Stumbrys, 2025). The importance of such continuing interdisciplinary efforts should not be underestimated (Koch, 1999, Chapter 5; Slaney, 2017).

## Data Availability

Research materials (e.g., questionnaires) are available upon request to interested researchers. Ethical concerns and privacy considerations (see University of Alberta Faculty of Arts, Science, and Law Research Ethics Board; Protocol ATEE 454) precluded extended retention of the original data (e.g., dream reports, questionnaire responses).
