# Supplementary material for: From living metaphors in impactful dreams to sublime disquietude and sublime enthrallment
Source: Front Psychol. 2026 Aug 11;17:1818552. doi: 10.3389/fpsyg.2026.1818552 (PMC13503208; doi:10.3389/fpsyg.2026.1818552)
Supplement: Supplementary file 1 [file Data_sheet_1.pdf]

A Prototypic Transcendent Dream:

It may be helpful to examine the metaphoric structure of a prototypic transcendent dream (the Snake Dream from Kuiken, 1995, p. 129-130):

> I dream that I am climbing a stairs, coming to a landing. There are windows on both sides [and] high walls. It's very bright. Four people are there, and one person in particular is ... interacting with me. He hands me a box that is gift-wrapped and ... about shoe size. I start opening the box and then I [see it has] a cover [with] snakes [on it] ... Obviously there is a feeling of friendship between us or a feeling of comfortable joking ... and I ask, "Oh, you wouldn't dare, would you?" And, he said, "Well, you'll have to open it and find out." // So I open the box and, when I open the box, this multitude, this large number of snakes come out of the box. They're very thin, and they're long like ribbons. They're black, shiny, with yellow heads. They all wrap around my lower right leg like a cuff ... I'm trying to push them away, to kick them off, feeling panicky, trying to ... pry them off. And the more I try, the more I'm getting nowhere, the tighter they are wrapping themselves -- // until I suddenly calm down and start looking at them and see how they fit together and how they are not dangerous snakes. I start looking at them and I notice the color of their backs and how they seem to be so quiet and peaceful. So, I start touching them gently and, as I'm touching them, I'm talking to them. And, eventually I say, "OK, now you have to leave," and they do leave... // When the snakes did leave, I felt like I was light, I was lighter in weight, but there was also a sense of release ...

In this dream, successive episodes (separated here by //) provide semantically resonant representations through which the dreamer becomes more respectfully aware of the dangerous beauty of the snakes' participation in distributed ontological intersubjectivity. In the first

episode, the snakes' presence is suggested by a pictorial image on the cover of a box that contains them; metaphorically their sentience is a teasingly hidden gift. In the second episode, the colorfully present snakes are disclosed in a threatening form that motivates the dreamer's anxious resistance; metaphorically their sentience is antagonistic. In the third episode, the same colorfully presented snakes are quiet and peaceful, motivating the dreamer's touch and dialogue; metaphorically their sentience is that of mutual intentionality. The snakes share the subjectivity of the dreamer; metaphorically their awareness—and the dreamer's—is mutual and respectful. The culminating moment of "release" is not appreciation of a gift or relief from antagonistic threat—but rather nonutilitarian regard. The snakes retain their beauty (as in the decorated box); they retain their danger (they still "must leave"); but they also have the capacity for dialogue. Like the Borges lines, like the Hotel Dream, each successive metaphor deepens and enlivens without erasing its predecessor.

Additional Findings:

Construct Validity: Study 1

Sublime Disquietude: Convergent Validity. Although our index of Sublime Disquietude has content validity, we also found evidence of its convergent validity. We assessed a hierarchical regression model that included (a) a scale that is conceptually related to Inexpressible Realizations (i.e., Evocative Imagery [Kuiken et al., 2012]; entered in the first step) and (b) two scales conceptually related to Self-Perceptual Depth (i.e., Intra-Dream Self-Reflection [Lee and Kuiken, 2015] and Existential Awareness [adapted from Batson and Schoenrade, 1991; see also Kuiken et al., 2006]; these variables were entered in the second step). The first step in this model was significant, $F(1,145) = 72.868$, $p < .001$, as was the second, $F(2, 143) = 43.210$, $p < .001$. Roughly, the Inexpressible Realizations component of Sublime Disquietude (e.g., "I sensed something that I could not find a way to express") converged with Evocative Imagery (e.g., "Some of the images that came to mind seemed pregnant with meaning"), $r^2 = .33$. Also, the Self-Perceptual Depth component of Sublime Disquietude (e.g., "I felt sensitive to aspects of my life I usually ignore") converged with the combination of Intra-Dream Self-Reflection (e.g., "I was aware of my body, my feelings, and the way I was thinking") and Existential Awareness (e.g., "I began to reconsider my existential convictions"), r^2 change $= .25$. In sum, sublime disquietude was associated with a linear combination of evocative imagery and existential self-awareness.

Sublime Enthrallment: Convergent Validity. Although our index of Sublime Enthrallment has content validity, we also found evidence of its convergent validity. For Sublime Enthrallment, we assessed a hierarchical regression model that included a conceptually related index of Inexpressible Realizations (i.e., Evocative Imagery; entered in the first step) and two

conceptually related indices of Spiritual Revitalization (i.e., Distributed Liveliness and Loss of Self-Boundaries, both adapted from the Mystical Experiences Scale [MES; Hood and Francis, 2013]; see also Kuiken et al., 2006); these variables were entered in the second step). The first step in this model was significant, $F(1,145) = 53.063$, $p < .001$, as was the second, $F(3, 143) = 56.698$, $p < .001$. In sum, the Inexpressible Realizations component of Sublime Enthrallment (e.g., "I sensed something that I could not find a way to express") converged with Evocative Imagery (e.g., "Some of the images that came to mind seemed pregnant with meaning"), $r^2 = .27$, and the Spiritual Revitalization component of Sublime Enthrallment (e.g., "I felt an inner freedom, a sense of liberation from life's tangles and hindrances") converged with the linear combination of Distributed Liveliness (e.g., "I had the sense that everything around me was somehow alive") and Loss of Self-Boundaries (e.g., "My sense of self did not have a clear boundary"), r^2 change = .32. In sum, Sublime Enthrallment was associated with a global aesthetic judgment (evocative imagery) and independently with two scales that resemble components of a well-established measure of extraverted mysticism in the MES.

Construct Validity: Study 2

Sublime Disquietude: Convergent Validity. To provide further evidence of the convergent validity of our index of Sublime Disquietude, we again assessed a hierarchical regression model that included (a) a scale that is conceptually related to Inexpressible Realizations (i.e., Evocative Imagery [Kuiken et al., 2012]; entered in the first step) and (b) two scales conceptually related to Self-Perceptual Depth (i.e., Intra-Dream Self-Reflection [Lee and Kuiken, 2015] and Existential Awareness [adapted from Batson and Schoenrade, 1991; see also Kuiken et al., 2006]; these variables were entered in the second step). The first step in this model was significant, $F(1,172) = 57.111$, $p < .001$, as was the second, $F(3, 170) = 61.724$, $p < .001$. As in Study 1, the

Inexpressible Realizations component of Sublime Disquietude converged with Evocative Imagery, $r^2 = .25$. Also, the Self-Perceptual Depth component of Sublime Disquietude converged with the combination of Intra-Dream Self-Awareness and Existential Awareness, r^2 change $= .32$. Briefly, sublime disquietude was associated with a global aesthetic judgment (evocative imagery) and independently with existential self-awareness.

Sublime Enthrallment: Convergent Validity. Similarly, for Sublime Enthrallment, we assessed a hierarchical regression model that included a conceptually related index of Inexpressible Realizations (i.e., Evocative Imagery; entered in the first step) and two conceptually related indices of Spiritual Revitalization (i.e., Distributed Liveliness and Loss of Self-Boundaries; these variables were entered in the second step). The first step in this model was highly significant, $F(1,172) = 35.644$, $p < .001$, as was the second, $F(1,170) = 36.440$, $p < .001$. As in Study 1, the Inexpressible Realizations component of Sublime Enthrallment converged with Evocative Imagery, $r^2 = .17$, and the Spiritual Revitalization component of Sublime Enthrallment converged with the combination of Distributed Liveliness and the Loss of Self-Boundaries, r^2 change $= .25$. In sum, as in Study 1, Sublime Enthrallment was associated with a global aesthetic judgment (evocative imagery) and independently with two components of extraverted mysticism (Hood and Francis, 2013).
